# Additive interaction of diabetes mellitus and chronic kidney disease in cancer patient mortality risk

**DOI:** 10.1038/s41598-022-24466-1

**Published:** 2022-11-19

**Authors:** Seohyun Kim, Gyuri Kim, Jae Hyeon Kim

**Affiliations:** 1 Department of Clinical Research Design and Evaluation, Samsung Advanced Institute for Health Sciences and Technology (SAIHST), Sungkyunkwan University, Seoul, 06355 Republic of Korea; 2 Division of Endocrinology and Metabolism, Department of Medicine, Samsung Medical Center, Sungkyunkwan University School of Medicine, 81, Irwon-Ro, Gangnam-Gu, Seoul, 06351 Republic of Korea

**Keywords:** Cancer, Endocrinology, Medical research, Nephrology, Oncology, Risk factors

## Abstract

We investigated the additive interaction of diabetes mellitus (DM) and chronic kidney disease (CKD) on the risk of mortality in cancer patients and evaluated the impact of diabetic kidney disease (DKD) on mortality in cancer patients with DM. We retrospectively analyzed 101,684 cancer patients. A multivariable Cox regression model was used for assessing mortality risk. Relative excess risk due to interaction (RERI), attributable proportion (AP), and synergy index (SI) were used to evaluate the additive interactive effect. The adjusted hazard ratio (aHR, 95%CI) for mortality was significant for those with CKD alone (1.53, 1.39–1.68), DM alone (1.25, 1.2–1.3), and both CKD and DM (1.99, 1.84–2.17) compared to non-CKD and non-DM cancer patients. The additive interaction between CKD and DM was significant (RERI 0.22[95%CI = 0.01–0.42], AP 0.11[0.01–0.21], SI 1.28[1.01–1.62]). Among cancer patients with DM, the presence of DKD raised the aHR for mortality (1.55, 95%CI = 1.33–1.81) compared to those without DKD. Coexistence of DM and CKD at the time of cancer diagnosis was significantly associated with an increased risk of mortality, and their interaction exerted an additive interactive effect on mortality. DKD was significantly associated with an increased risk of mortality in cancer patients with DM.

## Introduction

In view of the rapidly increasing global epidemic of diabetes mellitus (DM), previous studies have reported that DM is associated with an increased risk of developing cancer and cancer mortality in Asian as well as western populations^1–4^. Although cardiovascular disease was the major cause of death for people with DM in the UK in 2001, cancer overtook cardiovascular disease as the leading cause of death in 2018 for both men and women^5^. In addition, the presence of DM in cancer patients at the time of diagnosis was linked to an increased risk of all-cause mortality when compared to cancer patients without DM, according to a previous meta-analysis study with various cancer types^6^.

Chronic kidney disease (CKD) is associated with an increased risk of various cancers compared to normal kidney function^7,8^. In addition, people with CKD show a higher risk for cancer-specific mortality, as well as all-cause and cardiovascular mortality, compared to those without CKD in the general population^9,10^. Furthermore, among patients with cancer, CKD is also related to an elevated risk for all-cause and cancer mortality^11,12^. Considering that CKD develops as a microvascular complication in approximately one-half of patients with type 2 DM and one-third of patients with type 1 DM^13,14^, the association between the coexistence of DM and CKD and the risk of mortality in cancer patients has not been elucidated. Although a previous study reported that the coexistence of DM and CKD was associated with increased mortality of all-cause and cardiovascular mortality in western general populations^15^, there have been few studies exploring the relationship between the coexistence of DM and CKD and the risk of mortality in patients with cancer.

Therefore, the aim of the study was to evaluate the impact of DM and CKD on risk of all-cause mortality in cancer patients. We also assessed the additive interaction between DM and CKD in cancer patient mortality risk and explored the effect of the presence of diabetic kidney disease (DKD), as assessed by a reduction in eGFR or the presence of albuminuria, on the risk of mortality in cancer patients with preexisting DM.

## Results

### Baseline characteristics of the study population and mortality rate

All baseline characteristics are summarized in Table 1. The median follow-up duration was 4.35 years. Of the 14,143 all deaths and 502,981 person-years, the mortality rate (per 10,000 person-years) for subjects without both DM and CKD, subjects with CKD alone, DM alone, and both DM and CKD were 217.25, 684.22, 488.21, and 979.67, respectively. Mortality rate ratio compared to subjects without both DM and CKD was 3.15 (95% CI 2.87–3.45), 2.25 (95% CI 2.16–2.33), and 4.51 (95% CI 4.16–4.88) in subjects having CKD alone, DM alone, and both DM and CKD, respectively (Supplementary Table 1).

**Table 1:** Baseline characteristics.

	Non-DM		DM		P value
	Without CKD	With CKD	Without CKD	With CKD	
	(n = 78,665)	(n = 1734)	(n = 19,530)	(n = 1755)	
Age, years	55.2 ± 12.5	71.1 ± 9.7	63.1 ± 9.9	70.7 ± 8.7	< 0.001
**Sex**					< 0.001
Female	43,063 (54.7%)	544 (31.4%)	6608 (33.8%)	494 (28.1%)	
Male	35,602 (45.3%)	1190 (68.6%)	12,922 (66.2%)	1261 (71.9%)	
**BMI (kg/m**^**2**^**)**					< 0.001
Underweight (< 18.5 kg/m^2^)	2913 (3.7%)	57 (3.3%)	444 (2.3%)	42 (2.4%)	
Normal (18.5–22.9 kg/m^2^)	31,753 (40.4%)	531 (30.6%)	5747 (29.4%)	485 (27.6%)	
Overweight (23–24.9 kg/m^2^)	19,603 (24.9%)	482 (27.8%)	5218 (26.7%)	443 (25.2%)	
Obese (≥ 25 kg/m^2^)	24,396 (31.0%)	664 (38.3%)	8121 (41.6%)	785 (44.7%)	
**Alcohol Consumption**					< 0.001
Never	46,862 (59.6%)	1078 (62.2%)	10,445 (53.5%)	1019 (58.1%)	
Ever	13,022 (16.6%)	347 (20.0%)	4289 (22.0%)	414 (23.6%)	
Current	18,781 (23.9%)	309 (17.8%)	4796 (24.6%)	322 (18.3%)	
**Smoking Status**					< 0.001
Never	53,667 (68.2%)	1024 (59.1%)	10,436 (53.4%)	922 (52.5%)	
Ever	14,188 (18.0%)	479 (27.6%)	5436 (27.8%)	546 (31.1%)	
Current	10,810 (13.7%)	231 (13.3%)	3658 (18.7%)	287 (16.4%)	
**History of Hypertension**					< 0.001
No	59,886 (76.1%)	524 (30.2%)	9450 (48.4%)	290 (16.5%)	
Yes	18,779 (23.9%)	1210 (69.8%)	10,080 (51.6%)	1465 (83.5%)	
**Cancer Types**					< 0.001
Gastrointestinal	30,199 (38.4%)	717 (41.3%)	7834 (40.1%)	700 (39.9%)	
Urologic	5079 (6.5%)	368 (21.2%)	1553 (8.0%)	268 (15.3%)	
Gynecologic	1619 (2.1%)	17 (1.0%)	275 (1.4%)	15 (0.9%)	
Breast	18,145 (23.1%)	98 (5.7%)	1461 (7.5%)	76 (4.3%)	
Hepato-pancreatobiliary	4036 (5.1%)	111 (6.4%)	2656 (13.6%)	196 (11.2%)	
Lung	8786 (11.2%)	278 (16.0%)	4330 (22.2%)	374 (21.3%)	
Thyroid	8559 (10.9%)	46 (2.7%)	728 (3.7%)	37 (2.1%)	
Others	2242 (2.9%)	99 (5.7%)	693 (3.5%)	89 (5.1%)	

*Abbreviations: BMI Body Mass Index, CKD Chronic Kidney Disease, DM Diabetes Mellitus.

### Multivariable cox regression of all-cause mortality and additive interaction

**Figure 1:**
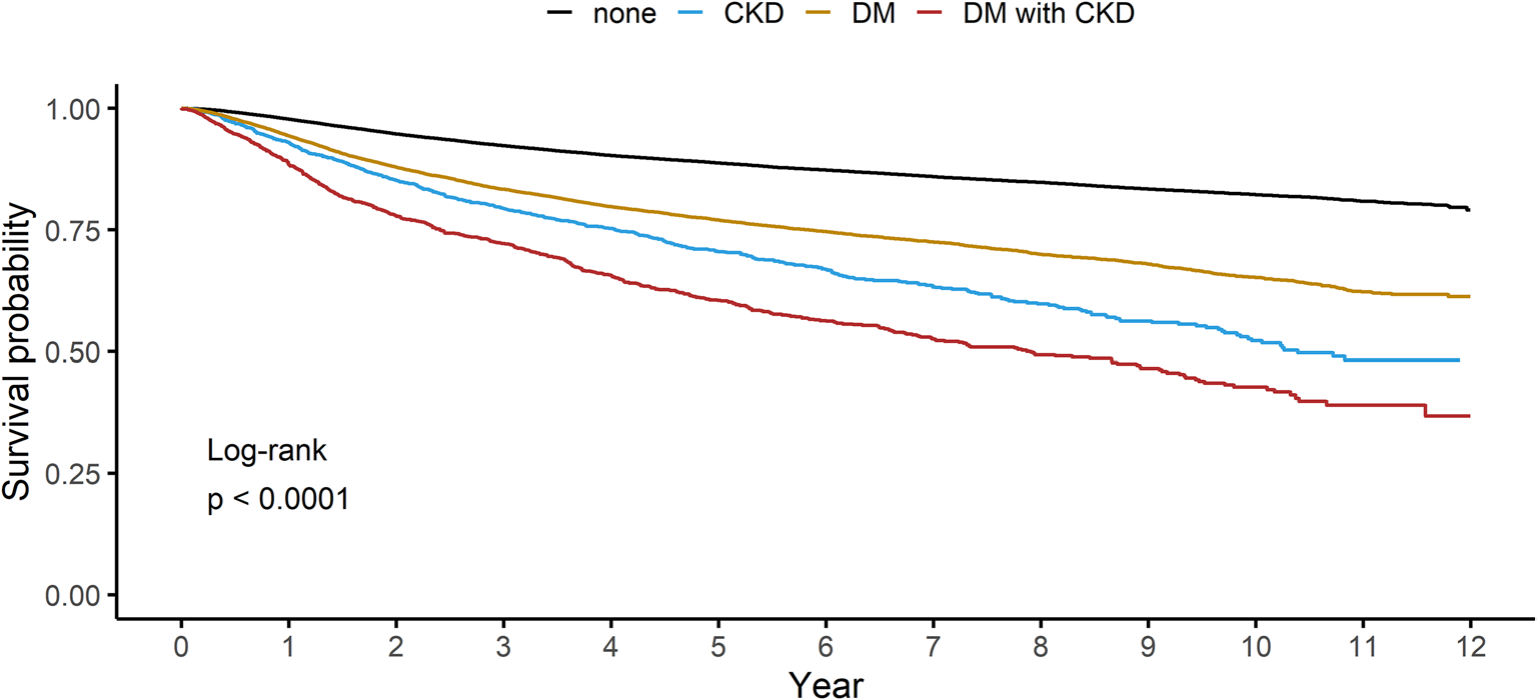
Kaplan–Meier estimates for all-cause mortality in cancer patients stratified by the presence of DM or CKD. *Abbreviations: CKD Chronic Kidney Disease, DM Diabetes Mellitus.

The unadjusted HRs for all-cause mortality in cancer patients with having CKD alone, DM alone, and both DM and CKD were 3.07 (95% CI 2.81–3.36), 2.21 (95% CI 2.13–2.30), and 4.36 (95% CI 4.02–4.72), compared to those without both CKD and DM, respectively (Table 2, Fig. 1). In multivariable analysis, CKD alone (adjusted HR [aHR] 1.53, 95% CI 1.39–1.68), DM alone (aHR 1.25, 95% CI 1.20–1.30), and both DM and CKD (aHR 1.99, 95% CI 1.84–2.17) were significantly associated with an increased risk of mortality compared to those without both CKD and DM. In subgroup analyses, there was no heterogeneous effect on the all-cause mortality depending on age, sex, and BMI (all P for interaction > 0.1) (Table 2, Supplementary Fig. 2 and 3). Although all parameters of the additive interaction effect were positive but not statistically significant in an unadjusted model (RERI 0.07, 95% CI -0.36 to 0.51; AP 0.02, 95% CI -0.08 to 0.12; SI 1.02, 95% CI 0.9–1.17; Table 2), they were statistically significant with positive values (RERI 0.22, 95% CI 0.01 to 0.42; AP 0.11, 95% CI 0.01 to 0.21; SI 1.28, 95% CI 1.01 to 1.62; Table 2) in the multivariable-adjusted model. When subjects were divided according to sex (male, female), age (< 60, ≥ 60), and BMI (< 25 kg/m^2^, ≥ 25 kg/m^2^), additive interaction was significant in females (RERI 0.65, 95% CI 0.14 to 1.16; AP 0.25, 95% CI 0.08 to 0.41; SI 1.66, 95% CI 1.11 to 2.48; Table 2) and subjects having BMI less than 25 kg/m^2^ (RERI 0.3, 95% CI 0.04 to 0.56; AP 0.15, 95% CI 0.03 to 0.26; SI 1.4, 95% CI 1.04 to 1.89).

**Table 2:** Multivariable Cox regression of all-cause mortality in cancer patients and additive interaction.

	Mortality Risk by exposure, HR (95% CI)				Additive Interaction (95% CI)		
	NO CKD or DM (n = 78,665)	CKD (n = 1734)	DM (n = 19,530)	DM and CKD (n = 1755)	RERI	AP	SI
Events	8726	504	4264	649			
Person-years	401,652	7366	87,339	6624			
Model 1	1 (Reference)	3.07 (2.81, 3.36)	2.21 (2.13, 2.30)	4.36 (4.02, 4.72)	0.07 (-0.36, 0.51)	0.02 (-0.08, 0.12)	1.02 (0.9, 1.17)
Model 2	1 (Reference)	1.53 (1.39, 1.68)	1.25 (1.2, 1.3)	1.99 (1.84, 2.17)	0.22 (0.01, 0.42)	0.11 (0.01, 0.21)	1.28 (1.01, 1.62)
**Sex**							
Male (n = 50,975)	1 (Reference)	1.46 (1.32, 1.63)	1.22 (1.16, 1.27)	1.84 (1.67, 2.03)	0.16 (-0.06,0.39)	0.09 (-0.03, 0.2)	1.24 (0.91, 1.67)
Female (n = 50,709)	1 (Reference)	1.66 (1.37, 2)	1.33 (1.23, 1.44)	2.63 (2.23, 3.11)	0.65 (0.14, 1.16)	0.25 (0.08, 0.41)	1.66 (1.11, 2.48)
*P* for interaction	*P* = 0.53						
**Age**							
< 60 (n = 55,757)	1 (Reference)	2.17 (1.54, 3.07)	1.42 (1.31,1.53)	2.58 (1.92, 3.48)	-0.01 (-1.07, 1.06)	0 (-0.42, 0.41)	1 (0.51, 1.95)
≥ 60 (n = 45,927)	1 (Reference)	1.86 (1.7, 2.05)	1.25 (1.2, 1.31)	2.26 (2.08, 2.47)	0.15 (-0.1, 0.4)	0.07 (-0.04, 0.17)	1.13 (0.92, 1.4)
*P* for interaction	*P* = 0.49						
**BMI**							
< 25 kg/m^2^ (n = 67,718)	1 (Reference)	1.51 (1.35, 1.69)	1.23 (1.17, 1.29)	2.04 (1.84, 2.26)	0.3 (0.04, 0.56)	0.15 (0.03, 0.26)	1.4 (1.04, 1.89)
≥ 25 kg/m^2^ (n = 33,966)	1 (Reference)	1.5 (1.27, 1.77)	1.24 (1.16, 1.33)	1.85 (1.6, 2.13)	0.1 (-0.24, 0.45)	0.06 (-0.13, 0.24)	1.14 (0.74, 1.76)
*P* for interaction	*P* = 0.51						

*Abbreviations: AP Attributable Proportion due to interaction, CI Confidence Interval, CKD Chronic Kidney Disease, DM Diabetes Mellitus, HR Hazard Ratio, RERI Relative Excess Risk due to Interaction, SI Synergy Index.

*Null hypothesis for each interaction is RERI = 0, AP = 0, and SI = 1.

*Model 1 is the unadjusted model; Model 2 is adjusted for age, sex, BMI, smoking status, alcohol consumption, history of hypertension, and cancer types.

*P for interaction was tested by three-way interaction.

### Association between DKD and risk of mortality in cancer patients with preexisting DM

**Figure 2:**
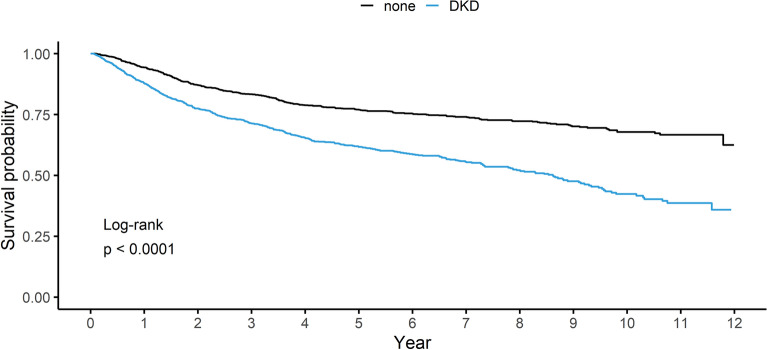
Kaplan–Meier curves for all-cause mortality in cancer patients with preexisting DM. *Abbreviations: DKD Diabetic Kidney Disease.

We evaluated the association between DKD and the risk of mortality in cancer patients with preexisting DM. Cancer patients with DKD were significantly associated with increased risk of all-cause mortality, compared to those without DKD (*p* < 0.001) (Fig. 2). In multivariable analysis, the unadjusted and adjusted HRs for all-cause mortality in cancer patients with DKD were 2.02 (95% CI 1.75–2.34), 1.55 (95% CI 1.33–1.81), respectively, compared to those without DKD (Table 3). In subgroup analysis by sex, aHRs of male and female for the risk of mortality with DKD were 1.51 (95% CI 1.27–1.8) and 1.7 (95% CI 1.22–2.37) without interaction in subgroups (p for interaction > 0.1). For further stratified analysis by the presence or absence of CKD (defined as eGFR < 60 ml/min/1.73 m^2^) or albuminuria (defined as UACR ≥ 30 mg/g), aHR of CKD and albuminuria group, albuminuria alone group, and CKD alone group was 1.77 (95% CI 1.47–2.15), 1.45 (95% CI 1.20–1.75), and 1.36 (95% CI 1.03–1.79), respectively, compared to those without both CKD and albuminuria group (Supplementary Table 2).

**Table 3:** Mortality rates and multivariable Cox regression of all-cause mortality in cancer patients with preexisting DM.

	Subjects (n)	Events (n)	Person-years	Mortality Rate	Model 1 (95% CI)				Model 2 (95% CI)			
					Overall	Male	Female	*P* for interaction	Overall	Male	Female	*P* for interaction
None	1,271	305	6,889	442.75	1 (Reference)	1 (Reference)	1 (Reference)	0.15	1 (Reference)	1 (Reference)	1 (Reference)	0.37
DKD	1,113	444	4,734	937.96	2.02 (1.75, 2.34)	1.85 (1.56, 2.18)	2.42 (1.79, 3.28)		1.55 (1.33, 1.81)	1.51 (1.27, 1.8)	1.7 (1.22, 2.37)	

*Abbreviations: BMI Body Mass Index, DM Diabetes Mellitus, DKD Diabetic Kidney Disease.

*Mortality rate was calculated by dividing death cases by 10,000 person-years.

*Model 1 is the unadjusted model; Model 2 is adjusted for age, sex, BMI, smoking status, alcohol consumption, history of hypertension, and cancer types.

*P for interaction was tested by two-way interaction.

### Sensitivity analysis

We further perform sensitivity test for cancer patients who achieved no-evidence-of-disease (NED) status with follow-up to the starting date of NED instead of continuing their follow-up to the end of the study. As shown in Supplementary Table 3, CKD alone (adjusted HR [aHR] 1.51, 95% CI 1.35–1.68), DM alone (aHR 1.22, 95% CI 1.17–1.27), and both DM and CKD (aHR 2.02, 95% CI 1.84–2.23) were significantly associated with an increased risk of mortality compared to those without both CKD and DM and all parameters of the additive interaction effect analysis were statistically significant. Also, in cancer patients with preexisting DM, DKD was significantly associated with increasing the mortality rate when censoring was considered for NED status (aHR 1.6, 95% CI 1.33–1.91, Supplementary Table 3).

## Discussion

In this large-scaled longitudinal study with 502,981 person-years, we demonstrated that the coexistence of DM and CKD as well as DM alone and CKD alone at the diagnosis of cancer was associated with increased risk of all-cause mortality in cancer patients. We identified a positive synergistic interactive relationship between DM and CKD on the risk of mortality in cancer patients, particularly in females and subjects having a BMI of less than 25 kg/m^2^ after adjusting for demographic variables, comorbidities and types of cancer. We also demonstrated that DKD was a significant risk factor for all-cause mortality among cancer patients with preexisting DM. Furthermore, we found that coexistence of albuminuria and eGFR less than 60 ml/min/1.73 m^2^ in cancer patients with DM was associated with the increased risk of all-cause mortality.

The results of our study were consistent with the context of previous studies. Regarding the independent risk of CKD alone on the all-cause mortality, when compared with aHRs of 1.68 (95% CI 1.17–2.40)^16^, 1.62 (95% CI 1.46–1.79)^10^, 1.41 (95% CI 1.13–1.77)^12^, and 1.42 (95% CI 1.23–1.65)^9^ in patients having various comorbidities in previous studies, the present study showed an aHR of 1.53 (95% CI 1.39–1.68). Our study found the risk of DM alone for all-cause mortality in cancer patients showing aHR of 1.25 (95% CI 1.20–1.30), which was comparable with aHR of 1.41 (95% CI 1.28–1.55) in the previous meta-analysis of 23 longitudinal studies^6^. Although a previous study showed an additive interaction between CKD and DM in the general population by adjusted difference in cumulative incidence^15^, we used the Cox proportional hazard model to show various parameters of the additive interaction between CKD and DM in cancer patients on the risk of mortality. Considering that the global incidence of DM and CKD in patients with cancer is growing^2,8,13,17^**,** clinicians should check laboratory results for DM and CKD in patients who were diagnosed with cancer without a history of DM and CKD. Moreover, given that DKD showed a significant association with mortality risk in cancer patients with preexisting DM, examination of albuminuria may be helpful for predicting clinical prognosis in patients with DM at the diagnosis of cancer. Also, since there was a synergetic effect of DM and CKD on mortality, patients should pay attention to control and prevent modifiable common risk factors of DM and CKD such as obesity, smoking, hypertension, dyslipidemia, and poor glycemic control when they are diagnosed with cancer^18^. In sensitivity analysis considering the status of NED, as a result, in cancer patients who achieved NED status, the additive interaction was still statistically significant and the magnitude of effect was greater than when NED status was not considered. In addition, when considering NED, the effect of DKD on mortality in cancer patients with preexisting DM was also statistically significant and more predominant than previous analyses. These results indicated that this association is more predominant for patients with persistent cancer status compared to when all cancer patients with NED status were included.

Several mechanisms have been implicated in the pathophysiology underlying the additive interaction of CKD and DM on mortality. First, hyperinsulinemia and hyperglycemia in cancer patients with both DM and CKD accelerate cancer development and progression^19,20^. A worse prognosis for cancer may result from hyperinsulinemia and insulin-like growth factor levels, which can promote the growth of cancer cells^20–22^. Acute exposure to hyperglycemia may also enhance endothelial cell permeability and increase metastasis risk by increasing the production of reactive oxidant species and altering the structural integrity of the basement membrane^23^. Second, inflammation and hypercoagulation, which are more aggravated in patients with hyperglycemia and renal insufficiency, may contribute to an increasing risk of mortality^24,25^. Glucose variability and renal dysfunction are associated with oxidative stress, endothelial dysfunction, and inflammatory and procoagulant genes and biomarkers^11,24^. In terms of DKD, these conditions are also associated with the presence of albuminuria, which is a strong independent risk factor for several metabolic diseases and cardiovascular disease^26^. Third, cancer patients who already had DM or CKD have a worse prognosis for responding to cancer treatment, including a higher risk of infection and perioperative mortality^6,11^. Particularly, after transarterial chemoembolization (TACE), patients with CKD are more likely to develop contrast nephropathy^27^ and patients with CKD may have limited treatment options. Finally, people with DM or CKD frequently also have other cardiovascular risk factors and can be more vulnerable to mortality compared to those without DM and CKD^28–30^. Future research is needed to identify biological mechanisms of the additive interaction effect between DM and CKD on risk of mortality in cancer patients.

The strength of our study is that it is the first study to evaluate the association between the coexistence of DM and CKD and all-cause mortality with long follow-up duration in a large sample size of Asian population and assess additive interaction between DM and CKD on death. In addition, it was also the first study to identify the effect of DKD, defined as having CKD or albuminuria, on the risk of mortality in cancer patients with preexisting DM. We conducted subgroup analyses to identify interaction effects of CKD and DM by sex, age, and BMI and DKD by sex. Finally, our research can be helpful in medical field in two ways. The results of the study could be used to reduce mortality by paying attention to the diagnosis of diabetes and chronic kidney disease carefully when cancer is first diagnosed. In addition, since cancer accompanied by kidney disease in patients with diabetes increases the mortality rate, clinicians can inform patients with diabetes the importance of glycemic management to prevent chronic kidney disease. Our study has several limitations. First, the study is based on a sample of Korean individuals and the single-center hospital-based population is not representative of all cancer patients, which could have potential selection bias. This study was conducted in one of the largest tertiary general hospitals in South Korea. Second, although we conducted the multivariable analysis adjusted for multiple confounders, the probability of residual confounding including cancer treatment could not be ruled out. Third, we used patient self-reporting for history of DM as one of the ways to define DM, which can cause misclassification bias in the definition of diabetes. Fourth, in this retrospective cohort study, because our cohort included newly diagnosed cancer patients, we could not compare the results to general population. Further studies are needed to compare these associations between general population and cancer patients.

We revealed that coexistence of DM and CKD at the time of cancer diagnosis is associated with increased risk of mortality in cancer patients. We showed additive interaction of DM and CKD on mortality risk after controlling for confounders. In addition, we found that among cancer patients with preexisting DM, DKD, a reduction in eGFR, or albuminuria were substantial risk factors for all-cause mortality.

## Methods

### Study design and population

The study population consisted of cancer patients aged more than 20 who visited the Samsung Medical Center (SMC), Seoul, Republic of Korea from January 2008 to December 2019. A total of 138,956 subjects who were diagnosed with cancer for the first time (International Classification of Disease, 10th revision; (ICD-10), C code) and had records with TNM stage were enrolled. We stratified those cancer patients according to the presence or absence of DM and CKD. We excluded subjects with no medical history of DM based on self-report, or no laboratory test results of hemoglobin A1c (HbA1c) or fasting glucose (n = 15,150). Subjects who had previous Type 1 DM with an ICD-10 code of E10 (n = 30), subjects with missing values for serum creatinine (n = 2520), who had kidney cancer (n = 3442), who had distant metastasis (n = 9031), and who had missing variables of BMI, history of alcohol consumption, smoking and hypertension (n = 7099) were excluded. Finally, a total of 101,684 subjects were analyzed (Supplementary Fig. 1).

For further analysis to evaluate the effect of DKD on the risk of mortality in cancer patients with DM, subjects who had laboratory results of urinary albumin to creatinine ratio (UACR) were included among the cancer patients with preexisting DM group (n = 2384).

### Definition of exposure

Data were extracted from the Clinical Data Warehouse (CDW) DARWIN-C of SMC for this study. Personal medical history including DM, hypertension, smoking history, alcohol consumption, and medication was assessed by electrical medical records and self-administered questionnaire. All subjects’ demographic, anthropometric, and laboratory data were collected. DM was defined as having E11-14 of ICD-10 codes, a self-reported history of DM, records of prescription for anti-diabetic medications, a 6.5% or higher in HbA1c, or 126 mg/dL or higher in fasting glucose. The value of estimated glomerular filtration rate (eGFR) was calculated using the Chronic Kidney Disease Epidemiology Collaboration 2021 (CKD-EPI 2021) formula^31^. The definition of presence of CKD was eGFR < 60 ml/min/1.73 m^2^ at the diagnosis of cancer^32^. DKD was defined as eGFR < 60 ml/min/1.73 m^2^ or UACR ≥ 30 mg/g at the diagnosis of cancer in patients with DM^13^.

### Definition of covariates

Body weight and height were measured and body mass index (BMI) was calculated as body weight (kg) divided by height squared (m^2^). Smoking status and alcohol consumption were collected by self-reported questionnaire and categorized as never, ever, or current. The history of hypertension was defined as having a self-reported history with hypertension, having I10-15 in ICD-10 codes, having records of prescriptions for anti-hypertensive medications, or having at least 3 times more than 140 mmHg in systolic blood pressure or 90 mmHg in diastolic blood pressure. According to primary site of cancer, we divided all cancers into 24 common categories^33^, and reclassified them into 8 cancer types including gastrointestinal (colon, rectum, stomach, esophagus, and small intestine), urologic (bladder, prostate, testis, and ureter), gynecologic (endometrial, cervix uteri, corpus uteri, and ovary), breast, hepato-pancreatobiliary (liver and intrahepatic bile duct, gallbladder and other parts of the biliary tract, and pancreas), lung, thyroid cancer, and other cancers^11,12^.

### Definition of outcomes

All patients were followed up from the date of their first diagnosis of cancer to the end of the study (December, 2019) or death (collected from the death records of SMC CDW linked to Statistics Korea).

### Statistical analysis

All continuous variables were presented as the mean and standard deviation (SD) and all categorical variables were presented as proportions. Analysis of variance (ANOVA) for continuous variables and the chi-square test for categorical values were used to assess the characteristics according to the presence of DM or CKD. Survival curves were analyzed by Kaplan–Meier’s method and compared with the log-rank test. We assessed hazard ratios (HRs) with 95% confidence interval (CI) for all-cause mortality using Cox proportional hazard regression models. To conduct multivariable analysis, we adjusted for age, sex (male and female), BMI (< 18.5 kg/m^2^, 18.5–22.9 kg/m^2^, 23–24.9 kg/m^2^,  ≥ 25 kg/m^2^), alcohol consumption (never, ever, current), smoking status (never, ever, current), history of hypertension (yes, no), and cancer type (gastrointestinal, urologic, gynecologic, breast, hepato-pancreatobiliary, lung, thyroid cancer and others). Then, we performed additive interaction analyses of DM and CKD on the risk of mortality by estimating additive interaction parameters, including relative excess risk due to interaction (RERI), attributable proportion due to interaction (AP), and synergy index (SI)^34–37^. We identified the additive interaction stratified by sex (male, female), age (< 60,  ≥ 60), and BMI (< 25 kg/m^2^,  ≥ 25 kg/m^2^). We confirmed the three-way interaction effect of CKD and DM with each subgroup and the two-way interaction effect of DKD and sex in cancer patients with preexisting DM. Finally, we conducted a sensitivity analysis to test the robustness of our study. Considering cancer patients who achieved no-evidence-of-disease (NED) status, which was determined by the oncologists during follow-up, they were censored at the date of starting NED instead of continuing follow-up to the end of the study^38^.The statistical significance was regarded as *P* < 0.05 and all analyses were conducted using R studio version 1.4.

### Ethics approval and consent to participate

The Institutional Review Board (IRB) of Samsung Medical Center approved this study (approval no. SMC 2021-08-092). An informed consent exemption was granted by the IRB because all data provided by the CDW of SMC to researchers were de-identified and released for research purposes. All methods were conducted in accrordance with Declarations of Helsinki.

## Supplementary Information

Supplementary Information 1.

Supplementary Information 2.

Supplementary Information 3.

## Data Availability

All relevant data are available in this article and supplementary files.

## References

[1] Tsilidis, K. K., Kasimis, J. C., Lopez, D. S., Ntzani, E. E. & Ioannidis, J. P. Type 2 diabetes and cancer: umbrella review of meta-analyses of observational studies. *BMJ (Clinical research ed.)* **350**, g7607, 10.1136/bmj.g7607 (2015). 10.1136/bmj.g7607 25555821

[2] Noto H, Tsujimoto T, Noda M (2012). Significantly increased risk of cancer in diabetes mellitus patients: A meta-analysis of epidemiological evidence in Asians and non-Asians. J. Diabet. Investig..

[3] Chen Y (2017). Association between type 2 diabetes and risk of cancer mortality: a pooled analysis of over 771,000 individuals in the Asia Cohort Consortium. Diabetologia.

[4] Pearson-Stuttard J (2021). Type 2 diabetes and cancer: An umbrella review of observational and mendelian randomization studies. Cancer Epidemiol., Biomark. Prev.: Publ. Am. Assoc. Cancer Res., Cosponsored Am. Soc. Prev. Oncol..

[5] Pearson-Stuttard J (2021). Trends in predominant causes of death in individuals with and without diabetes in England from 2001 to 2018: an epidemiological analysis of linked primary care records. Lancet Diabetes Endocrinol..

[6] Barone BB (2008). Long-term all-cause mortality in cancer patients with preexisting diabetes mellitus: a systematic review and meta-analysis. JAMA.

[7] Wong G (2009). Association of CKD and cancer risk in older people. J Am Soc Nephrol.

[8] Malyszko J, Tesarova P, Capasso G, Capasso A (2020). The link between kidney disease and cancer: Complications and treatment. Lancet (London, England).

[9] Tonelli M (2006). Chronic kidney disease and mortality risk: A systematic review. J Am Soc Nephrol.

[10] Weng PH (2011). Cancer-specific mortality in chronic kidney disease: longitudinal follow-up of a large cohort. Clin. J. Am. Soc. Nephrol.: CJASN.

[11] Na SY (2011). Chronic kidney disease in cancer patients: An independent predictor of cancer-specific mortality. Am. J. Nephrol..

[12] Ishii T (2020). Association between chronic kidney disease and mortality in stage IV cancer. Int. J. Clin. Oncol..

[13] Thomas MC (2015). Diabetic kidney disease. Nat. Rev. Dis. Primers..

[14] Targets G (2021). Standards of medical care in diabetes-2021. Diabetes Care.

[15] Afkarian M (2013). Kidney disease and increased mortality risk in type 2 diabetes. J. Am. Soc. Nephrol..

[16] Russo E (2021). Kidney disease and all-cause mortality in patients with COVID-19 hospitalized in Genoa, Northern Italy. J. Nephrol..

[17] Hwangbo Y (2018). Incidence of diabetes after cancer development: A korean national cohort study. JAMA Oncol..

[18] Jiang W (2020). Establishment and validation of a risk prediction model for early diabetic kidney disease based on a systematic review4m and meta-analysis of 20 cohorts. Diabetes Care.

[19] Richardson LC, Pollack LA (2005). Therapy insight: Influence of type 2 diabetes on the development, treatment and outcomes of cancer. Nat. Clin. Pract. Oncol..

[20] Abe R, Yamagishi S (2008). AGE-RAGE system and carcinogenesis. Curr. Pharm. Des..

[21] Rajpathak SN (2009). The role of insulin-like growth factor-I and its binding proteins in glucose homeostasis and type 2 diabetes. Diabetes Metab. Res. Rev..

[22] Pollak M (2008). Insulin and insulin-like growth factor signalling in neoplasia. Nat. Rev. Cancer.

[23] Morss AS, Edelman ER (2007). Glucose modulates basement membrane fibroblast growth factor-2 via alterations in endothelial cell permeability. J. Biol. Chem..

[24] Klimontov, V. V., Saik, O. V. & Korbut, A. I. Glucose Variability: How Does It Work? *Int. J. Mol. Sci.* **22**, 10.3390/ijms22157783 (2021). 10.3390/ijms22157783 PMC8346105 34360550

[25] Shlipak MG (2003). Elevations of inflammatory and procoagulant biomarkers in elderly persons with renal insufficiency. Circulation.

[26] Gansevoort RT (2013). Chronic kidney disease and cardiovascular risk: epidemiology, mechanisms, and prevention. Lancet (London, England).

[27] McCullough PA (2006). Risk prediction of contrast-induced nephropathy. Am. J. Cardiol..

[28] Li S, Wang J, Zhang B, Li X, Liu Y (2019). Diabetes mellitus and cause-specific mortality: A population-based study. Diabetes Metab. J..

[29] Lee MJ, Ha KH, Kim DJ, Park I (2020). Trends in the incidence, prevalence, and mortality of end-stage kidney disease in South Korea. Diabetes Metab. J..

[30] Lee S-H, Han K, Kwon H-S, Kim MK (2021). Frequency of exposure to impaired fasting glucose and risk of mortality and cardiovascular outcomes. Endocrinol Metab.

[31] Inker LA (2021). New creatinine- and cystatin c-based equations to estimate GFR without race. N. Engl. J. Med..

[32] K/DOQI clinical practice guidelines for chronic kidney disease: evaluation, classification, and stratification. *Am. J. Kidney Dis.: The Off. J. Nat. Kidney Foundation* **39**, S1-266 (2002). 11904577

[33] Jung KW, Won YJ, Kong HJ, Lee ES (2018). Prediction of cancer incidence and mortality in Korea, 2018. Cancer Res. Treat..

[34] de Mutsert R, Jager KJ, Zoccali C, Dekker FW (2009). The effect of joint exposures: examining the presence of interaction. Kidney Int..

[35] Jang YJ (2021). Additive interaction of mid- to late-life depression and cerebrovascular disease on the risk of dementia: A nationwide population-based cohort study. Alzheimer's Res. Ther..

[36] de Jager DJ, de Mutsert R, Jager KJ, Zoccali C, Dekker FW (2011). Reporting of interaction. Nephron. Clin. Practice.

[37] Li R, Chambless L (2007). Test for additive interaction in proportional hazards models. Ann. Epidemiol..

[38] Bishop AJ (2015). Prognosis for patients with metastatic breast cancer who achieve a no-evidence-of-disease status after systemic or local therapy. Cancer.

