# Supplementary figures and images for: Additive interaction of diabetes mellitus and chronic kidney disease in cancer patient mortality risk
Source: Sci Rep. 2022 Nov 19;12:19957. doi: 10.1038/s41598-022-24466-1 (PMC9675792; doi:10.1038/s41598-022-24466-1)

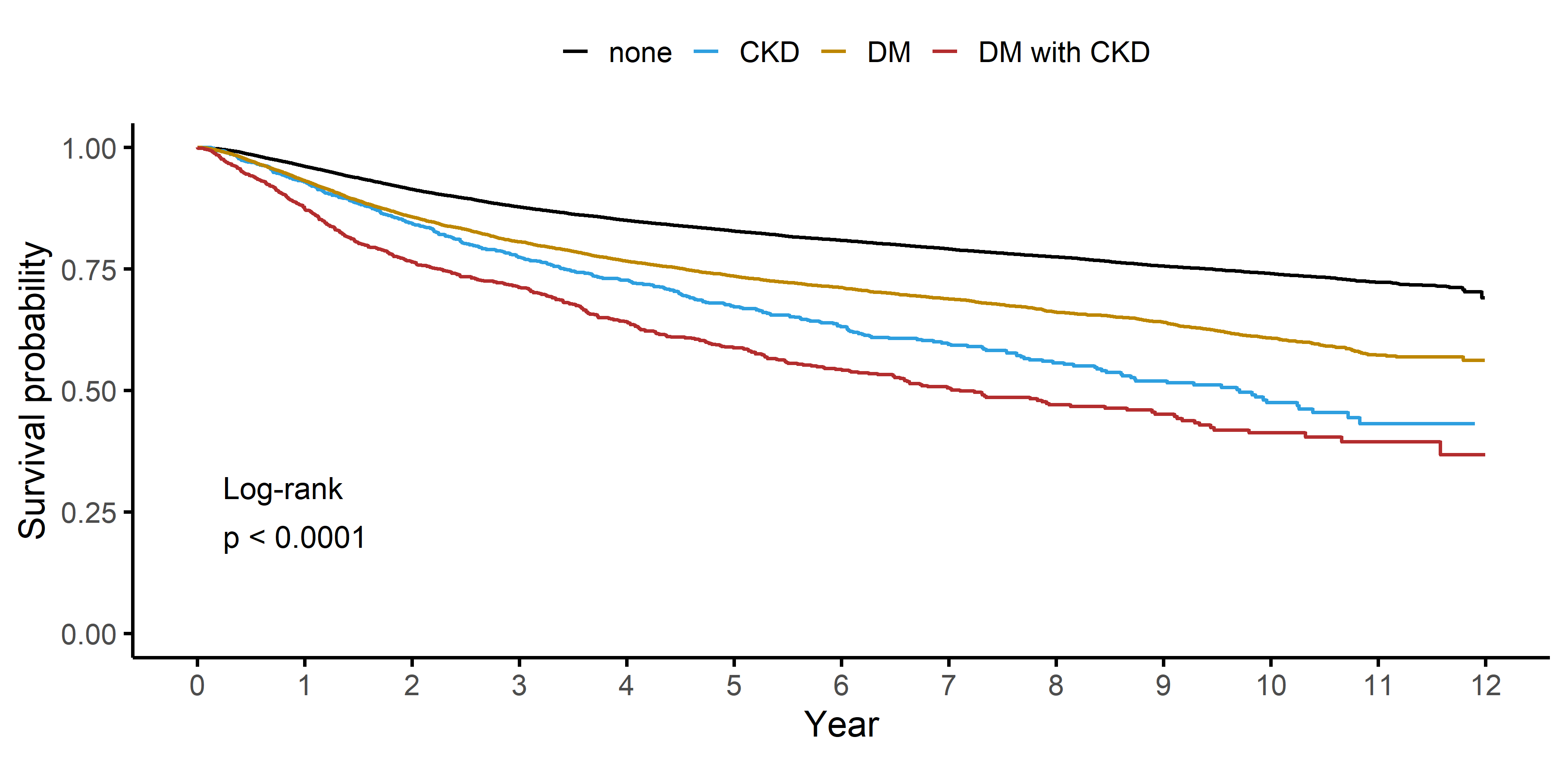

Supplement: Supplementary file 1 — Supplementary Information 1. [file 41598_2022_24466_MOESM1_ESM.tiff]

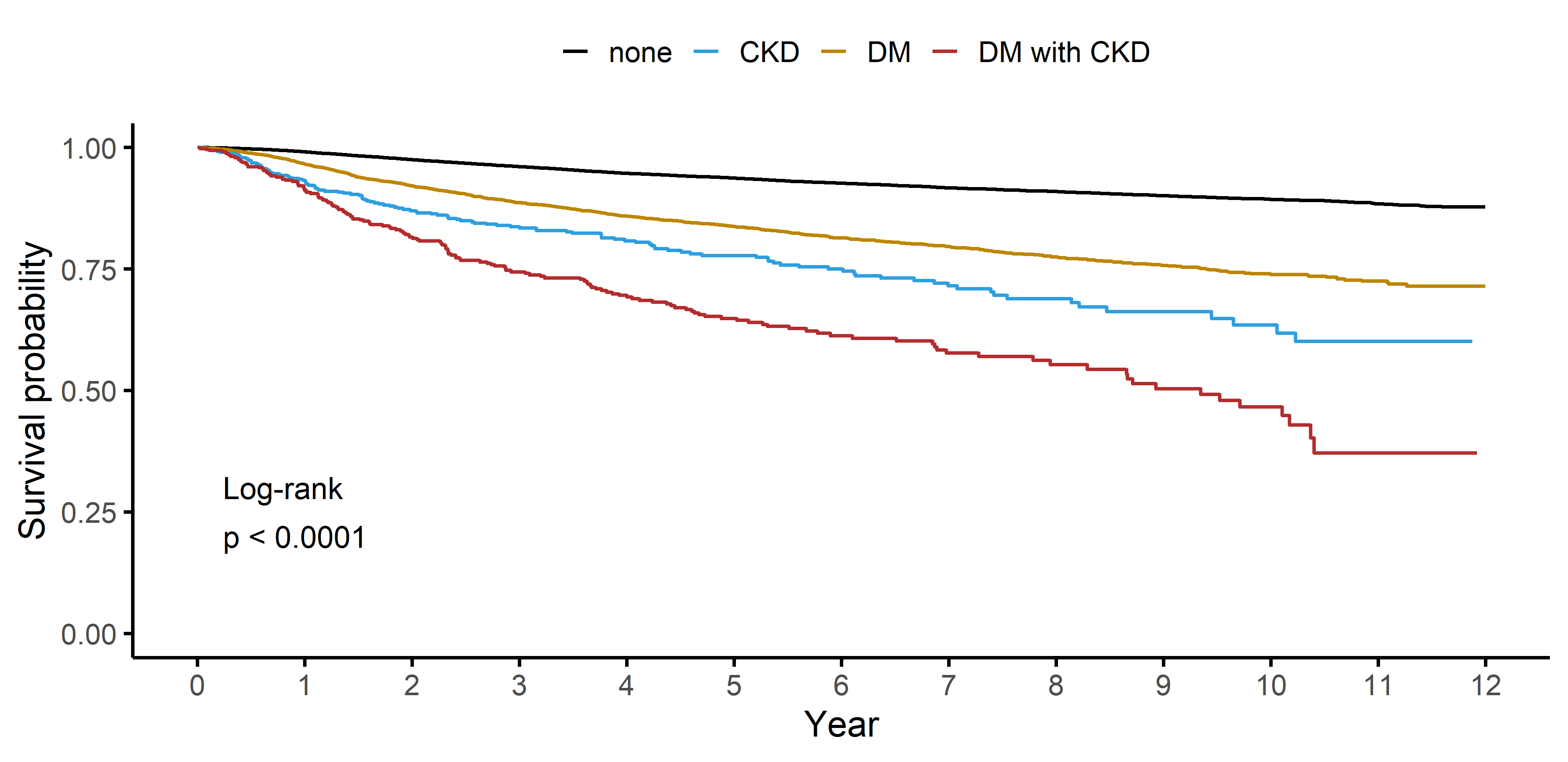

Supplement: Supplementary file 2 — Supplementary Information 2. [file 41598_2022_24466_MOESM2_ESM.tiff]
