# Supplementary material for: Additive interaction of diabetes mellitus and chronic kidney disease in cancer patient mortality risk
Source: Sci Rep. 2022 Nov 19;12:19957. doi: 10.1038/s41598-022-24466-1 (PMC9675792; doi:10.1038/s41598-022-24466-1)
Supplement: Supplementary file 3 — Supplementary Information 3. [file 41598_2022_24466_MOESM3_ESM.docx]

**Supplementary Material**

**Supplementary Table 1. Mortality rates in cancer patients.**

|  | **Subjects (n)** | | **Events (n)** | | **Person-years** | | **Mortality**  **Rate** | | **MRR (95% CI)** |
| --- | --- | --- | --- | --- | --- | --- | --- | --- | --- |
|  |  |  |  |  |  |  |  |  |  |
| **None** | | 78,665 | | 8,726 | | 401,652 | | 217.25 | 1 (Reference) |
| **CKD** | | 1,734 | | 504 | | 7,366 | | 684.22 | 3.15  (2.87, 3.45) |
| **DM** | | 19,530 | | 4,264 | | 87,339 | | 488.21 | 2.25  (2.16, 2.33) |
| **DM with CKD** | | 1,755 | | 649 | | 6,624 | | 979.67 | 4.51  (4.16, 4.88) |

*Abbreviations: CKD Chronic Kidney Disease, DM Diabetes Mellitus, MRR Mortality Rate Ratio

*Mortality rate was calculated by dividing death cases by 10,000 person-years, MRR (Mortality rate ratio) was estimated by ratio of mortality rate compared to patients without CKD and DM. aHRs (adjusted hazard ratios) were calculated with adjustments for age, sex, BMI classification, smoking status, alcohol consumption, history of hypertension, and cancer type.

**Supplementary Table 2. HRs of all-cause mortality in cancer patients with preexisting DM stratified by CKD and albuminuria.**

|  | **Without Albuminuria** | | **With Albuminuria** | |
| --- | --- | --- | --- | --- |
|  | **n** | **HR (95% CI)** | **n** | **HR (95% CI)** |
| **With or without CKD** |  |  |  |  |
| Without CKD  (eGFR ≥ 60) | 1,271 | 1.00 (*ref*) | 511 | 1.45  (1.20-1.75) |
| With CKD  (eGFR<60) | 165 | 1.36  (1.03-1.79) | 437 | 1.77  (1.47-2.15) |

*Abbreviations: BMI Body Mass Index, CI Confidence Interval, CKD Chronic Kidney Disease, DM Diabetes Mellitus, HR Hazard Ratio

*All models were adjusted for age, sex, BMI classification, smoking status, alcohol consumption, history of hypertension, and cancer type.

**Supplementary Table 3. Sensitivity Analysis Censoring the NED**

|  | **Subjects (n)** | | **Events (n)** | **Person-years** | **aHR (95% CI)** | **Additive Interaction (95% CI)** | | |
| --- | --- | --- | --- | --- | --- | --- | --- | --- |
|  |  |  |  |  |  | **RERI** | **AP** | **SI** |
| **Cancer patients with presence of CKD or DM** | | | | | | | | |
| **None** | | 78,665 | 6,746 | 255,714 | *ref* | 0.3 (0.05-0.54) | 0.15 (0.04-0.26) | 1.41 (1.06-1.89) |
| **CKD** | | 1,734 | 354 | 4,804 | 1.51  (1.35-1.68) |  |  |  |
| **DM** | | 19,530 | 3,292 | 57,552 | 1.22  (1.17-1.27) |  |  |  |
| **DM with CKD** | | 1,755 | 472 | 4375 | 2.02  (1.84-2.23) |  |  |  |
| **Cancer patients with preexisting DM stratified by DKD** | | | | | | | | |
| **None** | | 1,271 | 222 | 4,251 | *ref* |  |  |  |
| **DKD** | | 1,113 | 319 | 3,101 | 1.6  (1.33-1.91) |  |  |  |

*Abbreviations: aHR adjusted Hazard Ratio, AP Attributable Proportion due to interaction, CI Confidence Interval, CKD Chronic Kidney Disease, DM Diabetes Mellitus, DKD Diabetic Kidney Disease, RERI Relative Excess Risk due to Interaction, SI Synergy Index

*All the aHRs mentioned above are adjusted for age, sex, BMI, smoking status, alcohol consumption, history of hypertension, and cancer type.

**Supplementary Figure 1. Flow chart**

First cancer diagnosis from 2008-01-01 to 2019-12-31 by TNM code

(age ≥ 20)

N = 138,956

N = 101,684

(non-DM population = 80,399 / DM population = 21,285)

Analysis for DKD

N = 2,384

**Exclusion**

Missing data for urine albumin creatinine ratio in cancer patients with DM (n=18,901)

**Exclusion**

1. Missing data for history of diabetes (n=15,150)

2. Having previous E10 (Type 1 diabetes) codes (n=30)

3. Missing data for serum creatinine (n=2,520)

4. Kidney cancer (n=3,442)

5. Having distant metastasis (n=9,031)

6. Missing data for covariates (n=7,099)

-Missing data for smoking status (n=5,672)

-Missing data for alcohol consumption (n=1,036)

-Missing data for BMI (n=377)

-Missing data for hypertension (n=14)

*Abbreviations: BMI Body Mass Index, CKD Chronic Kidney Disease, DM Diabetes Mellitus, DKD Diabetic Kidney Disease

**Supplementary Figure 2. Kaplan-Meier curves for all-cause mortality in male cancer patients**

**
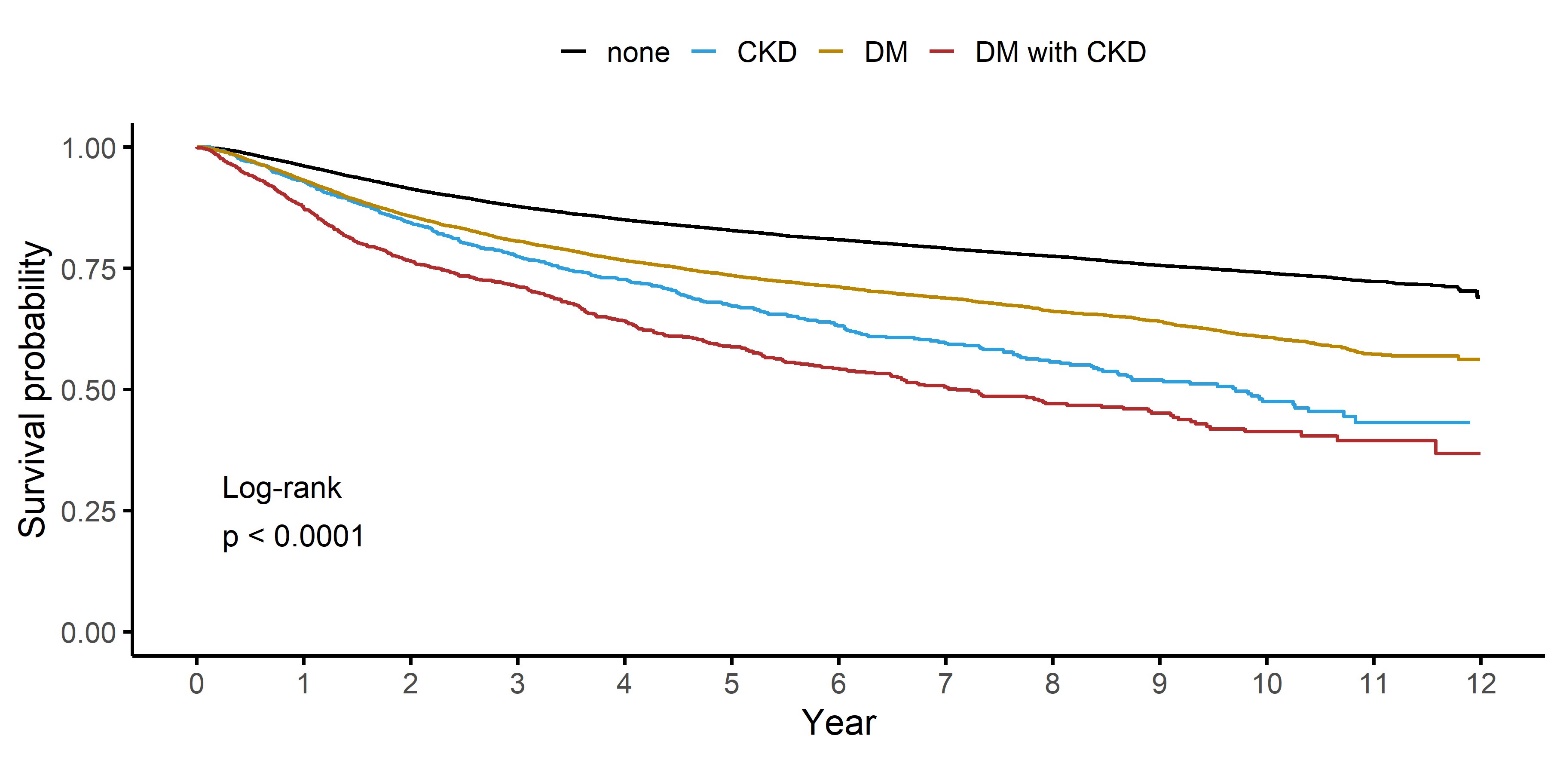
**

*Abbreviations: CKD Chronic Kidney Disease, DM Diabetes Mellitus

**Supplementary Figure 3. Kaplan-Meier curves for all-cause mortality in female cancer patients**

**
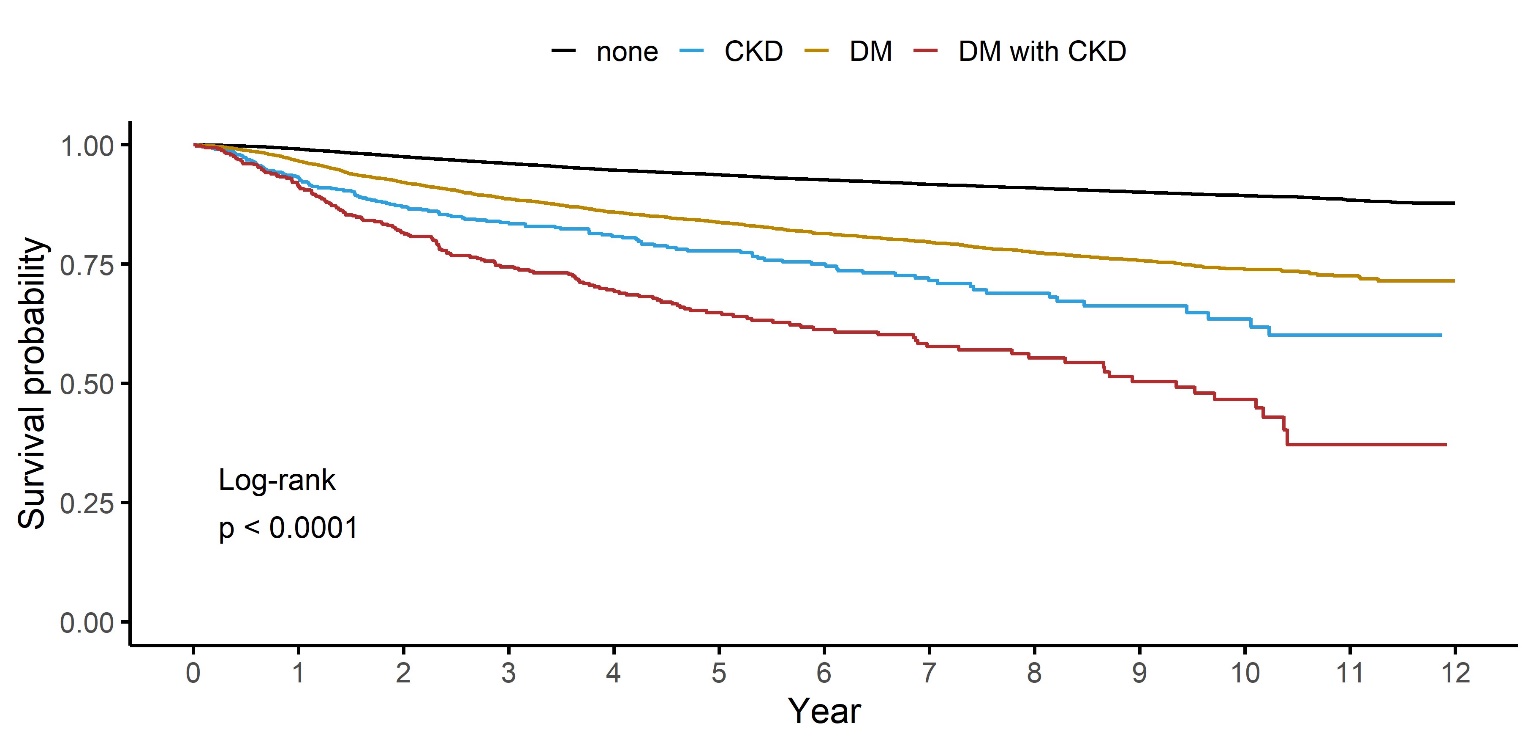
**

*Abbreviations: CKD Chronic Kidney Disease, DM Diabetes Mellitus
